# Supplementary material for: High Prevalence of Multidrug-Resistant Klebsiella pneumoniae in a Tertiary Care Hospital in Ethiopia
Source: Antibiotics (Basel). 2021 Aug 20;10(8):1007. doi: 10.3390/antibiotics10081007 (PMC8388856; doi:10.3390/antibiotics10081007)
Supplement: Supplementary file 1 [file antibiotics-10-01007-s001.zip › antibiotics-1183756-supplementary.pdf]

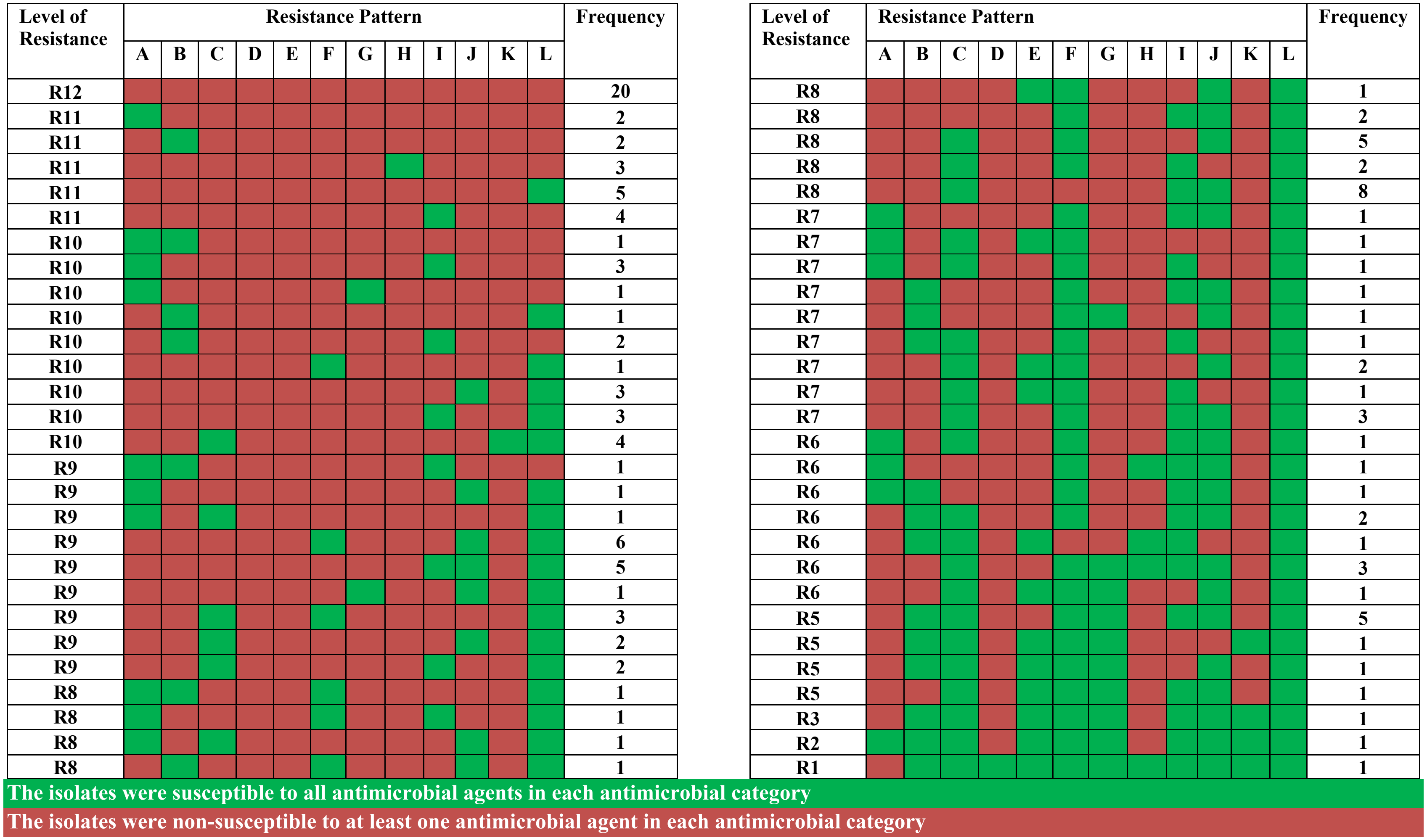

Figure S1. Multidrug resistance patterns of *K. pneumoniae* isolates.

Antimicrobial categories based on Magiorakos *et al.* (2012): — **A:** Tetracyclines, **B:** Aminoglycosides, **C:** Fluoroquinolones, **D:** 1st and 2nd generation cephalosporins, **E:** Monobactams, **F:** Antipseudomonal penicillins + β-lactamase inhibitors, **G:** Penicillins + β-lactamase inhibitors, **H:** Folate pathway inhibitors, **I:** Phenicols, **J:** Cephamycins, **K:** 3rd and 4th generation cephalosporins, **L:** Carbapenems

Antimicrobial Treatment History of Study Participants

In this study, data on the participant's history of antimicrobial treatment within the past 3 months were collected from their medical records. Based on this from the total study participants, 83 (62.9%) and 20 (15.2%) have taken 3rd or 4th generation cephalosporins and carbapenems, respectively.

Table S1. Previous antimicrobial treatment status of study participates (*n* = 132)

Categories of Antimicrobials	Yes *n* (%)	No *n* (%)
**Any Antimicrobial Treatment**	116 (87.9)	16 (12.1)
**Carbapenems**	20 (15.2)	112 (84.8)
**3rd or 4th Generation Cephalosporins**	83 (62.9)	49 (37.1)
**Quinolones**	14 (10.6)	118 (89.4)
**Aminoglycosides**	43 (32.6)	89 (67.4)
